# Post-operative infections after cardiothoracic surgery and vascular procedures: a bibliometric and visual analysis of the 100 most-cited articles in the past 2 decades

**DOI:** 10.3205/dgkh000484

**Published:** 2024-05-17

**Authors:** Mohsan Ali, Bisma Akram, Masooma Zainab Bokhari, Aleena Ahmed, Amar Anwar, Muhammad Talha, Rawal Alias Insaf Ahmed, Areeba Mariam Mehmood, Bisal Naseer

**Affiliations:** 1 King Edward Medical University, Lahore, Pakistan; 2 MBBS Scholar, King Edward Medical University, Lahore, Pakistan; 3 MBBS Scholar, Combined Military Hospital Medical College, Lahore, Pakistan; 4 Provincial Disease Surveillance & Response Unit, Hyderabad, Sindh, Pakistan; 5 Sargodha Medical College, Sargodha, Pakistan

**Keywords:** bibliometric analysis, visual analysis, cardiothoracic surgery, vascular surgery, surgical site infections

## Abstract

**Aim:**

To recognize and analyze the 100 most-cited articles on post-operative infections following cardiothoracic surgery and vascular procedures in the past 20 years.

**Methods:**

Articles published on post-operative infections following cardiothoracic surgery and vascular procedures from inception 1986 till 2020 were reviewed and selected by two authors, based on their number of citations using the Scopus database. Their characteristics were recorded, i.e., title, authors, publication date, total no. of citations, citations per year (CPY), country of research, institutional affiliation, journal, research subject, and article type.

**Results:**

The top 100 most influential articles were published between 1968 and 2017, with the peak in 2002. The mean number of total citations was 236.79 (range: 108–1,157). Areas with a medical focus were predominant in the studied research articles on the researched topic. The top-most journals in which these articles were published include Annals of Thoracic Surgery (14), followed by Circulation (8), and the New England Journal of Medicine (8). The number of publications affiliated with an institution were highest in the United States, with the Cleveland Clinic Foundation (6) having the most.

**Conclusion:**

These findings highlight that there is a great potential to conduct research and publish the prevalence, causes, risk factors, pathogenesis and molecular biology of post-cardiac and -vascular surgery infections to prevent their adverse effects. The results can be taken into consideration for policy making to improve post-cardiac-surgery outcomes.

## Zusammenfassung

**Zielsetzung:**

Erfassen und analysieren der 100 meist zitierten Artikel über postoperative Infektionen nach kardiothorakalen und vaskulären Eingriffen in den letzten Jahren.

**Methode:**

Artikel über postoperative Infektionen nach kardiothorakalen Operationen und vaskulären Eingriffen, die von 1986 bis 2020 veröffentlicht wurden, wurden von zwei Autoren anhand der Anzahl der Zitate in der Scopus-Datenbank gesichtet, ausgewählt, und ihre Merkmale erfasst wie Titel, Autoren, Veröffentlichungsdatum, Gesamtzahl der Zitate, Zitate pro Jahr, Land der Forschung, institutionelle Zugehörigkeit, Zeitschrift, Forschungsthema und Art des Artikels.

**Ergebnisse:**

Die 100 wichtigsten Artikel wurden zwischen 1968 und 2017 veröffentlicht, wobei der Höhepunkt im Jahr 2002 lag. Der Mittelwert der Gesamtzitationen betrug 236,79 (Spanne: 108–1.157). Bei den analysierten Artikeln überwiegen medizinisch ausgerichtete Bereiche. Zu den wichtigsten Zeitschriften, in denen die Artikel veröffentlicht wurden, gehören Annals of Thoracic Surgery (14), gefolgt von Circulation (8) und New England Journal of Medicine (8). Die Zahl der Veröffentlichungen, die mit einer Einrichtung verbunden sind, ist in den Vereinigten Staaten am höchsten mit der Cleveland Clinic Foundation (6) an der Spitze.

**Schlussfolgerung:**

Die Ergebnisse zeigen, dass ein großes Potenzial für die Erforschung der Prävalenz, Ursachen, Risikofaktoren, Pathogenese und Molekularbiologie von postoperativen Wundinfektionen nach herz- und gefäßchirurgischen Eingriffen besteht, um deren nachteilige Auswirkungen zu verhindern.

## Introduction

Cardiac surgeries pose a serious risk of surgical site infections (SSI) due to their highly invasive nature [1]. Although quantifying the incidence of infectious complications following cardiovascular surgeries is difficult, the reported rates of SSI range from 3.5%–26.8% [2], [3], [4], [5], [6]. Of all the SSI, deep sternal wound infections (DSWI) are the most dangerous [7]. These infections are significantly associated with mortality, increased cost of treatment, and lengthy hospital stays [8], [9].

An extensive range of risk factors come into play concerning SSI following major cardiothoracic surgeries. These include older age [10], increased body mass index, history of diabetes [11], smoking [12], use of grafts [13] and blood transfusion [14]. Effective implementation of patient optimization, optimal skin preparation, and proper surgical techniques are required to prevent post-operative infections, including DSWI [15], [16].

Given the ever-changing nature of microorganisms and the potentially devastating impact of SSI following cardiothoracic and vascular surgeries, there is a need to conduct a thorough analysis of available literature in this particular field to guide further research and policy-making. Bibliometric analysis is an effective, easy-to-implement, widely used technique for analyzing the literature [17] in a number of fields, including orthopedics, otorhinolaryngology, epidemiology, and pulmonology [18], [19], [20], [21].

This bibliometric and visual analysis focuses on the 100 most-cited articles on SSI following cardiothoracic and vascular surgeries. We analyzed the key characteristics of the selected top-cited articles, including an assessment of major research themes, authors’ collaboration, and key factors contributing to the increase in the number of citations. 

## Method

The bibliometric analysis was conducted using the Scopus database. Two reviewers searched the database in December 2023. The reviewers used the Scopus filters to separate original articles and review articles, and only original articles were selected for this bibliometric analysis. We included all articles related to SSI after cardiothoracic surgery and vascular procedures for which complete author information, including names, gender, and country of origin, were available. Articles for which author information or citations were unavailable were excluded, along with review articles and treatment guidelines. The reviewers assessed the relevance of the screened articles by a thorough appraisal of their abstracts. If abstracts were unavailable, other sources were used to find the abstracts and assess their appropriateness according to the inclusion criteria. To arrange the screened articles in order of citations, the reviewers used the “cited by” filter in Scopus. A list of 100 most-cited, original articles was ultimately compiled, and only mutually agreed-upon articles were included by both reviewers.

The Statistical Package for Social Sciences (SPSS) version 26 and Microsoft Excel version 2016 were used to analyze the final list of articles. Data were presented as tables and graphs using Microsoft Excel. 

## Results

The final list of the 100 most-cited publications is given in Table 1 (Tab. 1) in descending order of citations per year. Further, we also calculated the citations per year by dividing the number of citations of that particular article by the time since publication (in years). 

### Publication trends over time

**Figure 1:**
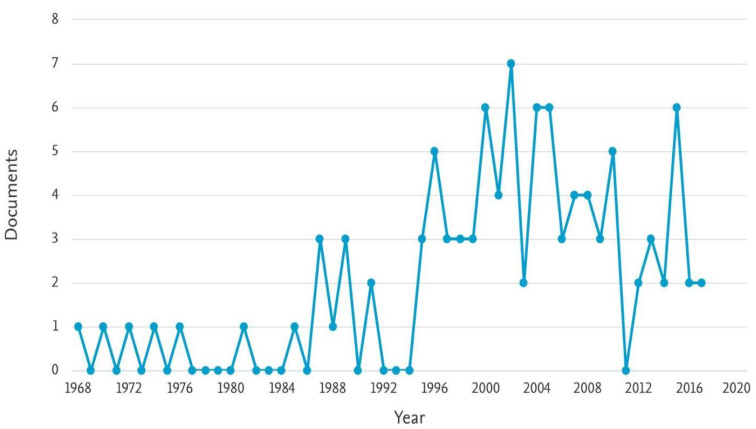
Trends in papers published per year

Intriguing patterns emerge from a temporal examination of the top 100 cited articles on SSI following vascular procedures and cardiothoracic surgery. A significant upsurge in publications took place in 2002 with seven publications. This was followed by six publications each in 2000, 2004, 2005, and 2015, indicating that these years were critical for the discipline (Figure 1 (Fig. 1)). This temporal progression implies ongoing research into new areas, which could be driven by developments in technology and new medicinal approaches. 

### Distribution across journals

**Figure 2:**
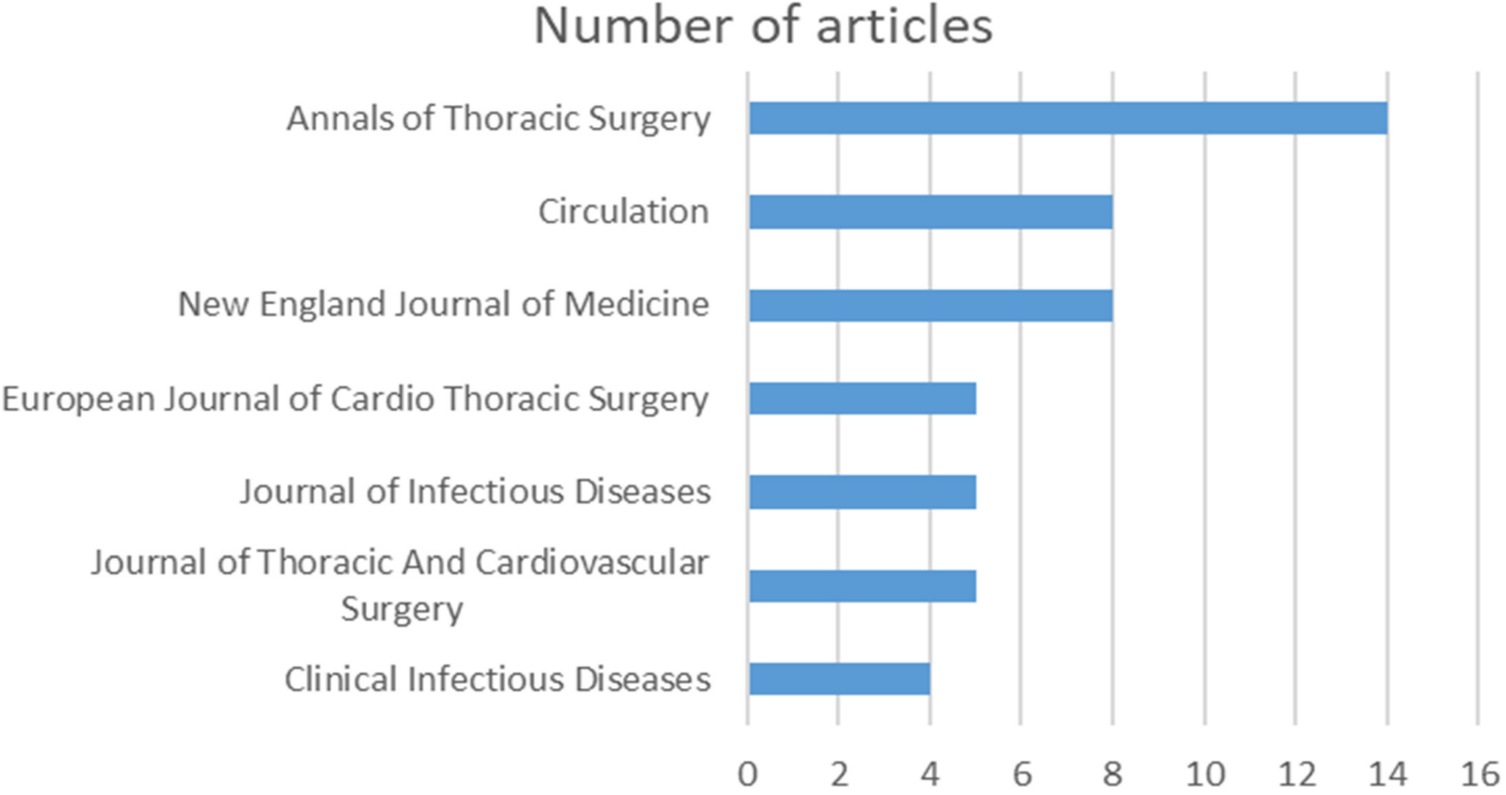
Journals with more than 3 publications

The way the publications are distributed among source titles highlights the channels that are favored for the dissemination of research on SSI following cardiothoracic surgery and vascular procedures. Annals of Thoracic Surgery takes the lead with 14 publications, followed by Circulation (8) and the New England Journal of Medicine (8), as shown in Figure 2 (Fig. 2). The implementation of this multifaceted distribution method advances a thorough comprehension of SSI following cardiothoracic surgery and vascular procedures. 

### Authorship patterns

Collaboration dynamics and important contributors can be identified by analyzing authorship patterns. Among the most prolific authors, Blackstone EH, Sax H, and Sommerstein R. stand out as noteworthy figures with four publications each (Table 2 (Tab. 2)). Several authors have contributed more than two articles, demonstrating a network of scholars sharing their experience. 

### Affiliation analysis

Institutional contributions are highlighted by conducting an extensive examination of affiliations. The Cleveland Clinic Foundation stands out with 6 publications, followed by the University of Zurich and University Spital Zurich with 4 publications each, indicating their centrality in this research (Table 3 (Tab. 3)). This is a global collaborative effort, with institutions from United States, United Kingdom, and Germany involved. The variety of affiliations enhances the field of research by providing different perspectives into the condition. 

### Country distribution

**Figure 3:**
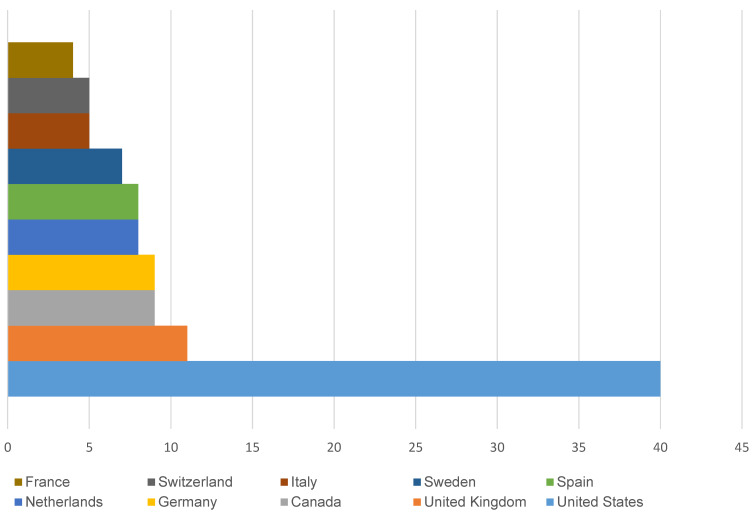
Countries with more than 3 publications

The geographic distribution of publications illustrates the worldwide reach of research concerning SSI following cardiothoracic surgery and vascular procedures. The United States leads with 40 publications, followed by the United Kingdom (11) and Canada (9), as shown in Figure 3 (Fig. 3). The field benefits from this international collaboration by assimilation of varied ideas and approaches. Research’s worldwide scope broadens the pool of knowledge and makes it easier to develop insights and interventions.

### Subject area distribution

**Figure 4:**
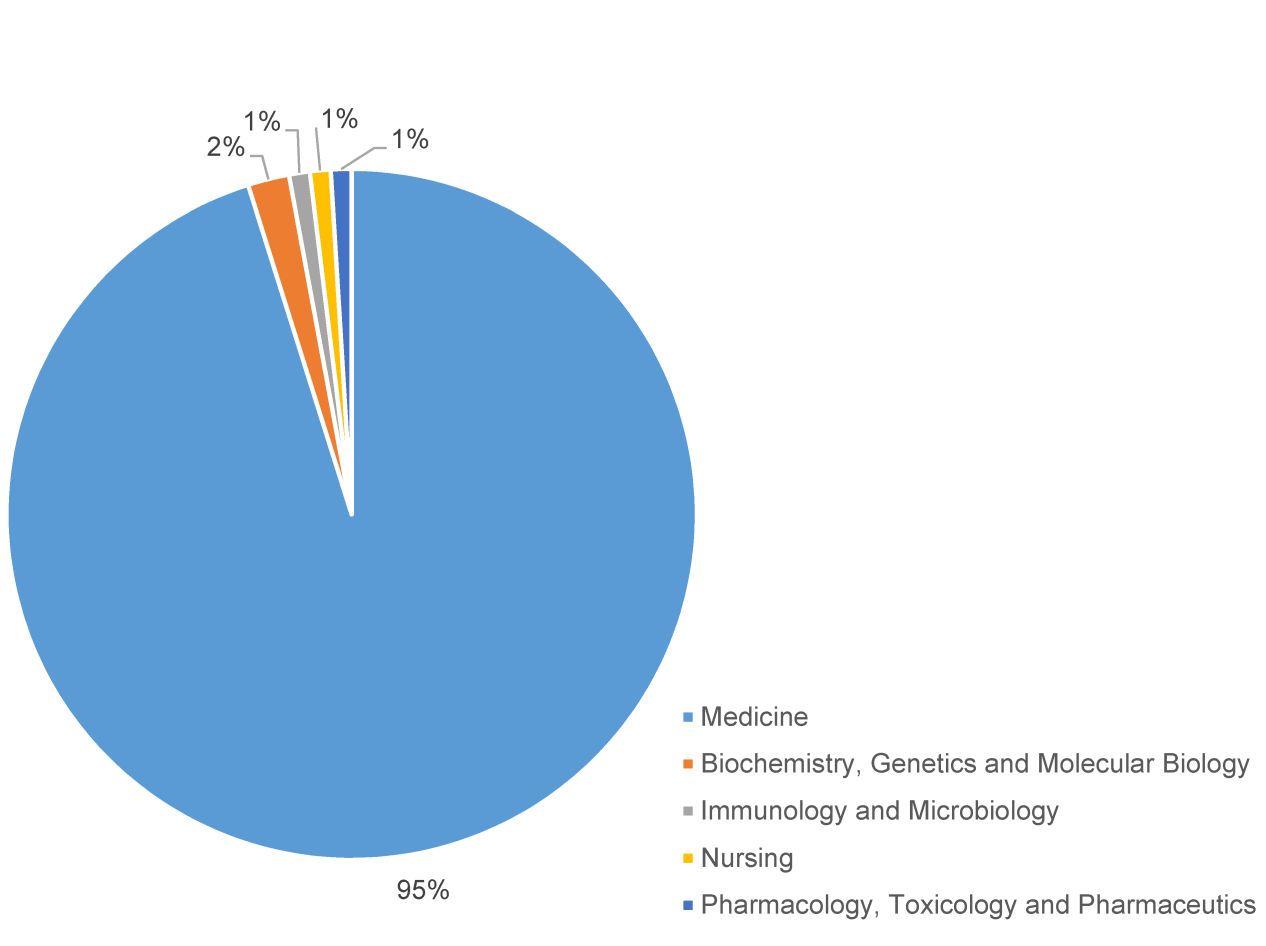
Subject area distribution

Research on SSI after cardiothoracic surgery and vascular procedures is interdisciplinary, as evidenced by a careful review of the topic areas with an emphasis on their clinical relevance, but the majority of articles are in the realm of medicine. Additional contributions reflect the collaborative character and wider significance of the research, with contributions in the fields of biochemistry, genetics and molecular biology, immunology and microbiology, nursing, pharmacology, toxicology, and pharmaceutics (Figure 4 (Fig. 4)). This multidisciplinary collaboration demonstrates a comprehensive strategy for understanding and managing post-operative infections following vascular and cardiothoracic surgeries.

## Discussion

Cardiothoracic surgery and vascular procedures are some of the most common surgical operations carried out globally, and due to their aggressive nature, they are linked to a high risk of SSI. A vast amount of literature on infections following cardiothoracic and vascular surgeries is contained in the databases, making it difficult for academics and physicians to locate pertinent articles quickly. They can locate their articles of interest using this bibliometric analysis, which will also benefit them in their clinical work.

Based on the number of citations, a bibliometric study entitled “Duration of red-cell storage and complications after cardiac surgery” ranked among the top 100 articles which had the greatest influence on the topic of SSI following cardiothoracic surgery and vascular operations. The citation count of a paper has become an important indicator in evaluating its importance [22], [23]. The articles in our analysis had citations per year ranging from 2.0 to 72.3.

Observational studies were the most prevalent type of research design (including case-control, cohort, cross-sectional, and epidemiological studies). Clinical trials were the second most popular type of article. There were only a small number of review articles and meta-analyses.

The distribution of publications among various journals revealed the preferred avenues for publicizing research. Only three journals – the Annals of Thoracic Surgery, Circulation, and the New England Journal of Medicine – contain 30 of the 100 publications included in this analysis. The remaining publications were distributed among other journals.

Some writers have submitted more than two publications, indicating a network of authors who are contributing valuable work in this field. A variety of viewpoints is highlighted by the synthesis of the authors’ contributions and trends. This collaborative approach enriches the literature by incorporating a wide range of expertise.

The study’s research articles clearly show institutional contributions. These institutional contributions and trends not only highlight the progress made in addressing postoperative infections but also provide insights into potential avenues for future research and institutional practices.

According to a temporal analysis of the top 100 cited articles, there was a notable increase in the number of publications between 2000 and 2005. Research activity peaked during this time, indicating a rising interest in and understanding of the significance of SSI in the fields of cardiothoracic and vascular operations. With six publications, 2015 stands out as yet another pivotal year in the temporal evolution. The temporal trends that have been observed suggest a dynamic evolution of the field of inquiry. 

The majority of the studies that were part of our study were conducted in the United States and Europe. The integration of different ideas and approaches from diverse geographical regions has broadened knowledge horizons. Through the utilization of diverse healthcare systems’ experience and data, researchers can customize interventions for particular contexts, considering regional variations in patient demographics, healthcare infrastructure, and infectious disease profiles.

According to the most-cited publication, by Koch et al. [24], longer red blood cell storage time is linked to more complications following thoracic surgery. These results indicate that transfusion of red blood cells for more than two weeks increases the likelihood of complications following surgery. Likewise, the second-most cited article, by Murphy et al. [25], demonstrated that greater morbidity and mortality, as well as higher rates of postoperative infections, are substantially linked to red blood cell transfusion. 

The most-studied topic, with a total of 19 papers, was the incidence, risk factors, and outcomes of SSI in cardiothoracic and vascular procedures. These papers address a variety of subjects, including epidemiology [26], mediastinitis [27], [28], [29], nasal Staphylococcus carriage [30], [31], an superficial as well as deep sternotomy site infections [32], [33]. Transfusion-related complications comprised the second most studied topic, with a total of ten papers. These articles included information on the following topics: outcomes of blood transfusions [34], [35], red blood cell transfusions [25], red blood cell storage duration [24], use of leukocyte reduction filters [36], [37], liberal versus restrictive transfusion [38], retrovirus transmission through transfusion [39], and the impact of blood conservation on surgical outcomes [40].

Infective endocarditis [41], [42], [43], obesity and diabetes [44], the role of glycemic control [45], [46], [47], [48], [49], the role of antibiotic prophylaxis, nosocomial infections, corticosteroid prophylaxis, antibiotic resistance, sources of contamination, management, infections related to grafts and devices, bleeding-related complications, inflammatory responses following cardiac operations, infections related to Hepatitis C and E, aspergillus infection, valve surgeries, and the economic aspects of postoperative infections are some other noteworthy topics.

Our research is valuable in several ways. By analyzing the top 100 cited articles on SSI following cardiothoracic surgery and vascular procedures, this study provides a diverse range of perspectives and insights from different regions across the globe. This study provides a dynamic evolution in the field of SSI during cardiac and vascular procedures. The temporal paradigm aids in the formation of a detailed understanding of the field’s past developments and present course. The international scope of SSI research can be seen by highlighting the top contributors, including the United States, the United Kingdom, and Canada. It also reveals a research gap in some regions of the world such as Asia and Africa. This lays the foundation for novel ideas and groundbreaking research, thus inspiring future investigations and advancements in the field that may eventually affect healthcare practice. The knowledge obtained from the most frequently referenced papers can help build evidence-based interventions targeted at reducing post-operative infections, as well as decision-making processes and research priorities.

## Conclusions

These findings highlight that there is a great potential to conduct research to propagate knowledge on the prevalence, causes, risk factors, pathogenesis and molecular biology of post cardiac and vascular SSI to prevent their adverse effects. The results can be taken into consideration for policy-making to improve post-cardiac-surgery outcomes.

### Limitations

The study had certain limitations. As only papers from the Scopus database were used for this analysis, impor-tant articles from other databases may have been neglected. One notable limitation of this study is its potential citation bias. The use of citation counts as a criterion for evaluation may overemphasize older publications, potentially ignoring the most recent ground-breaking contributions. Papers for which author information and citations were unavailable were excluded from this analysis, which may have erroneously reduced the significance of these papers. Finally, we did not analyze citations made in lectures, textbooks, or self-citations. Our results could have been influenced by inappropriate self-citations.

Despite these limitations, this study provides valuable insights into the top-cited literature on SSI following cardiothoracic surgery and vascular procedures. Recognizing these limitations is essential for accurately interpreting the findings and guiding future research in the field.

## Notes

### Authors’ ORCIDs

- Mohsan A: 0000-0002-5697-0458
- Rawal AIA: 0009-0009-7033-2354
- Bisma A: 0009-0005-8539-732X
- Masooma ZB: 0009-0005-7578-0827
- Amar A: 0000-0002-2371-6138
- Talha M: 0009-0006-1735-1247
- Bisal N: 0000-0002-9713-1255

### Competing interests

The authors declare that they have no competing interests.

## Figures and Tables

**Table 1:**
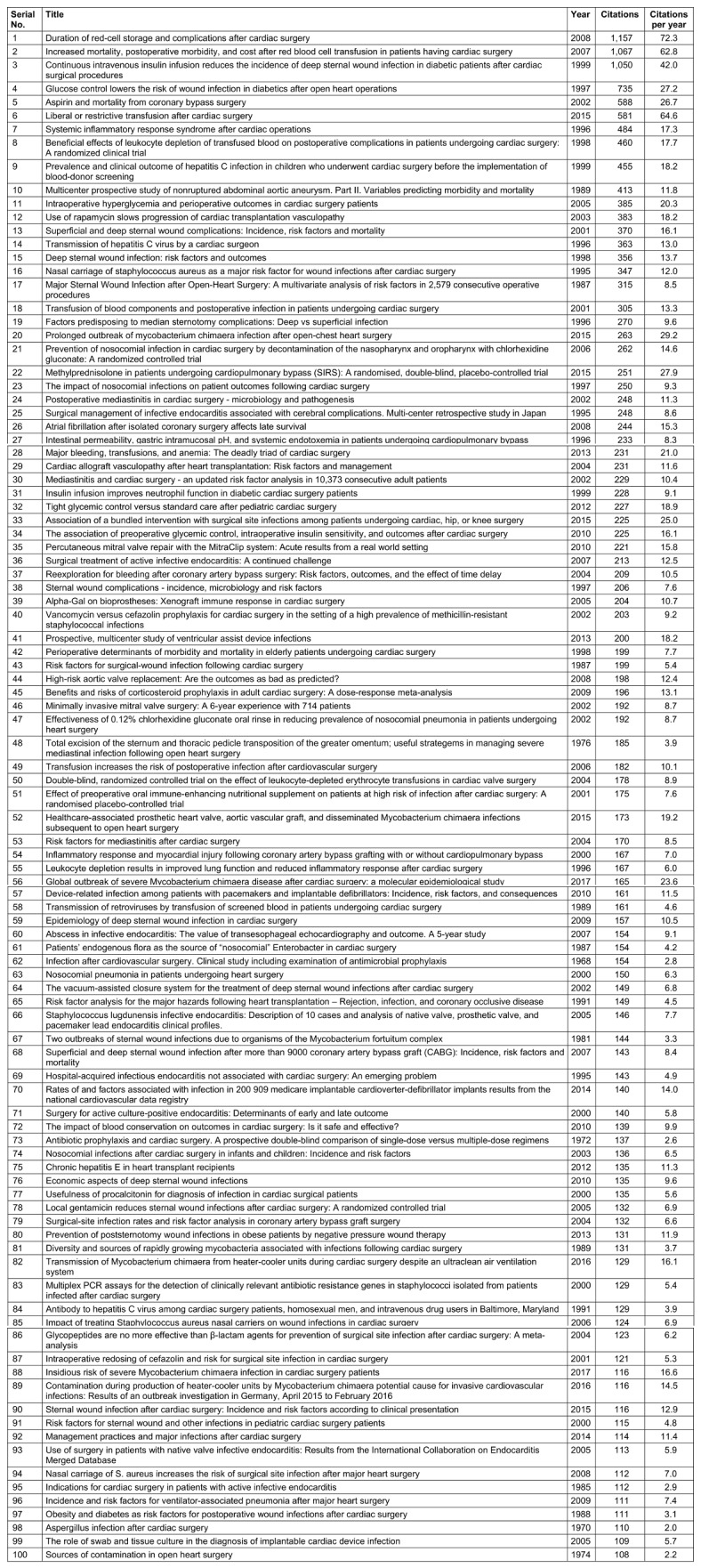
List of the top 100 most-cited publications in descending order of their citations

**Table 2:**
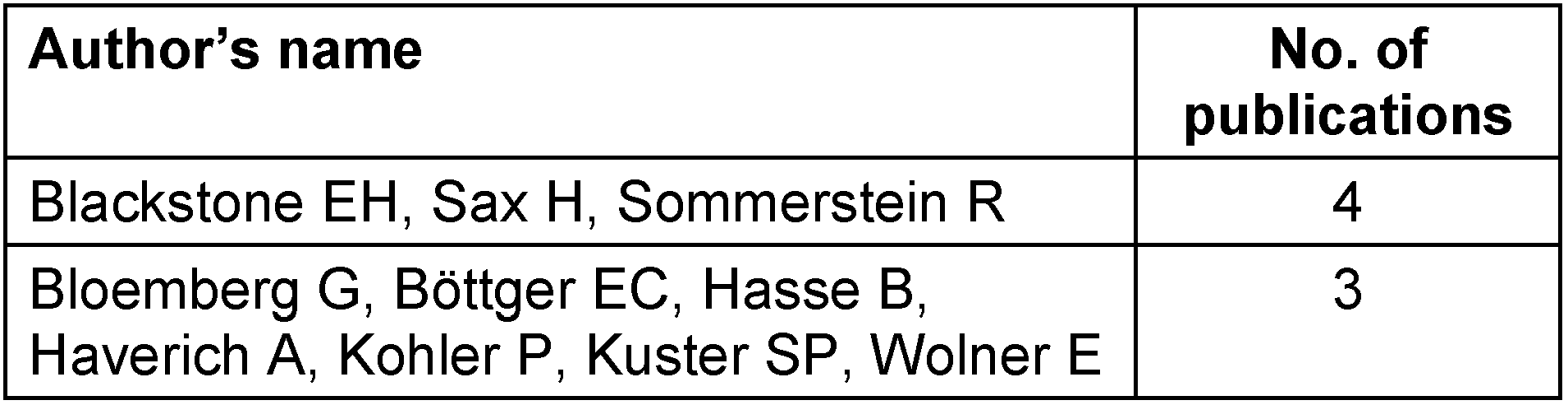
Authors with more than 2 publications

**Table 3:**
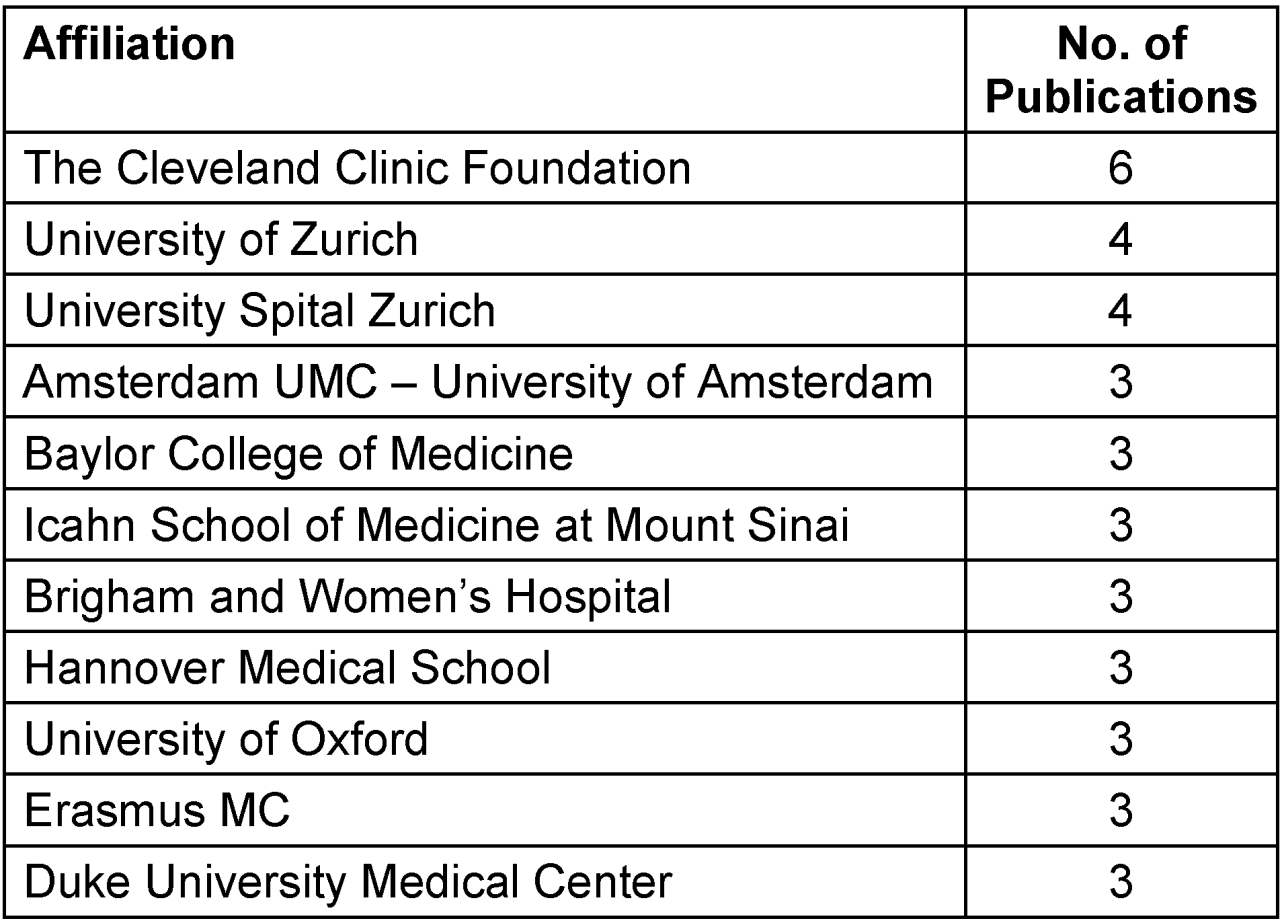
Institutions with more than 2 publications
